# Single-cell landscape of immunocytes in patients with extrahepatic cholangiocarcinoma

**DOI:** 10.1186/s12967-022-03424-5

**Published:** 2022-05-13

**Authors:** Lei Xu, Yun Lu, Zhengdong Deng, Xiangyu Li, Yuanxin Shi, Kai Zhao, Wei Yao, Jianming Wang

**Affiliations:** 1 Department of Biliary and Pancreatic Surgery/Cancer Research Center Affiliated Tongji Hospital, Tongji Medical College, Huazhong University of Science and Technology, Wuhan, 430030 Hubei China; 2 Department of Oncology Affiliated Tongji Hospital, Tongji Medical College, Huazhong University of Science and Technology, Wuhan, 430030 Hubei China; 3 Affiliated Tianyou Hospital, Wuhan University of Science & Technology, Wuhan, 430064 China

**Keywords:** Extrahepatic cholangiocarcinoma, Immunocyte, Single-cell RNA sequencing, Tumor heterogeneity, Cellular interactions

## Abstract

**Background:**

The intricate landscape of immunocytes in the tumor microenvironment (TME) is fundamental to immunotherapy but notably under-researched in extrahepatic cholangiocarcinoma (ECCA).

**Methods:**

Single-cell RNA sequencing technology was conducted to make an in-depth analysis of immunocytes from matched tumor tissues, paratumor tissues and peripheral blood from ECCA patients. The potential cellular interactions between two cell populations were analyzed with software CellPhoneDB (v2.1.7).

**Results:**

We obtained 13526 cells and characterized the transcriptomes and heterogeneity of different clusters and subclusters of immunocytes from ECCA, including CD4+ T cells, CD8+ T cells, B cells and myeloid immunocytes. We observed the rarely described immunocyte subclusters "intermediate" exhausted CD8+ T (CD8+ Tex) cells and “nonclassic” plasmacytes (CD27+ CD138+ CD38−). In addition, we identified potential immunotherapy targets, for example, ACP5, MAGEH1, TNFRSF9 and CCR8 for Tregs and MT1 for CD8+ Tex cells. We also found strong cellular interactions among Treg cells, M2 macrophages and CD8+ Tex cells through ligand–receptor analysis, implying that potential cellular cross-linkage promoted the immunosuppressive nature of the TME.

**Conclusions:**

In a word, our study illuminated the components of the TME and revealed potential cellular interactions at the individual cellular level in ECCA, we aimed to provide a new perspective for further immunological studies and immunotherapy of ECCA.

**Supplementary Information:**

The online version contains supplementary material available at 10.1186/s12967-022-03424-5.

## Introduction

Cholangiocarcinoma (CCA) is a malignant tumor originating from the epithelial cells of the biliary tree [1]. The incidence of CCA has steadily increased worldwide, and it currently ranks as the second most common hepatobiliary tumor [2], but the treatment of CCA treatment has recently shown embraced limited progression. It is widely known that CCA is characterized by a dense and reactive desmoplastic stroma around the cancer mass. Immunocytes are important components of the stroma and are closely related to prognosis [3]. Immunotherapies targeting immune checkpoint inhibition have achieved breakthroughs in melanoma and non-small-cell lung cancer. Checkpoint inhibitors have been reported for a small cohort of CCA patients but did not show the expected success [4]. Thus, a systematic investigation of the phenotype and function of immunocytes in CCA could help to map the immune environment and provide new strategies and targets for immunotherapy of cholangiocarcinoma.

Even traditional bulk-phase sequencing has revealed that driver mutations and aberrant regulation promote progression [5–7], and refined identification and functional studies in the field of immunocyte subclusters in the tumor microenvironment (TME) are still lacking. The application of single-cell RNA sequencing (scRNA-seq) to analyze the TME could illuminate the intricate immunocyte composition of the TME and the corresponding phenotypes and functions, particularly for low-abundance cell subpopulations. Recently, scRNA-seq has achieved breakthroughs in the study of various tumors, including lung cancer [8, 9], hepatocellular carcinoma [10, 11] and colorectal cancer [12]. Analysis of subclusters of mesenchymal cells at the single-cell level revealed the potential physiological functions of each subcluster and identified potential immunotherapy targets [10, 13]. Currently, there are few scRNA-seq data available for cholangiocarcinoma, and we could only find a report on intrahepatic cholangiocarcinoma [14]. However, scRNA-seq has never been used to study extrahepatic cholangiocarcinoma (ECCA).

Based on scRNA-seq, we captured data on 13526 single-cell transcriptomes to characterize the immune microenvironment of ECCA from the matched tumor tissues, paratumor tissues and peripheral blood of two ECCA patients. Our study clustered immunocytes, revealed immunocyte heterogeneity and described the transcriptome characteristics of different immunocyte clusters. We found potential immunotherapy targets, for example, *ACP5, MAGEH1, TNFRSF9 and CCR8* for tumor infiltrating Tregs and *MT1* for exhausted CD8+ T cells (CD8+ Tex cells). We also discovered some new subclusters, such as "intermediate" CD8+ Tex and “nonclassic” plasmacyte subclusters (*CD27*+ *CD138*+ *CD38*−). Moreover, we elucidated a potential cell-to-cell interaction network among Tregs, CD8+ Tex cells and M2 macrophages in ECCA.

## Methods

### Patients and clinical sample collection

Two ECCA patients who did not receive chemotherapy or radiation were enrolled in this study. Patients signed informed consent forms independently and agreed to donate tumor tissues and matched paratumor tissues and peripheral blood for scientific study. Our study was approved by the Tongji Hospital Research Ethics Committee and the Institutional Review Board. The demographic characteristics and clinical information of the patients researched by scRNA-seq are listed in Additional file 3: Table S1. The samples from two patients were collected at different times but by the same methods. After collection during surgery for ECCA, tumor and paratumor tissues were immediately transferred into MACS Tissue Storage Solution (Miltenyi Biotec GmbH), and peripheral blood was obtained before surgery. All fresh samples were stored on ice during transportation.

### Isolation of mononuclear cells

Tumor tissues and matched paratumor tissues were dissociated into single cells with a Human Tumor Dissociation Kit (Miltenyi Biotec GmbH) under the guidance of the kit instructions and resuspended in HBSS (Thermo Fisher Scientific) plus 0.04% bovine serum albumin (Sigma–Aldrich). Then, blood, tumor tissue and paratumor tissue, simultaneously, were processed by the same methods. Percoll separation solutions (Sigma–Aldrich) were adjusted to isotonicity and densities of 1.077 g/ml and 1.050 g/ml and were placed successively in tubes. The single-cell suspension obtained above and the matched blood mixed with an equal volume of PBS were laid on top of Percoll and centrifuged at 600×*g* for 30 min at 20 °C. Mononuclear cells, mainly lymphocytes and myeloid immunocytes, were collected from the third layer between two Percoll layers of different densities. Viability was confirmed to be> 85% in all samples with an AO/PI double staining kit (Thermo Fisher Scientific).

### ScRNA-seq library construction and sequencing

ScRNA-seq library construction was performed using the Chromium Single Cell 5′ Library & Gel Bead Kit (10× Genomics) for all samples according to the manufacturer’s instructions. Briefly, a single-cell suspension (containing 10000 cells), barcoded gel beads and partitioning oil were loaded onto Chromium Chip A to generate a single-cell gel bead-in-emulsion (GEM). Cells were captured in GEMs during targeted cell recovery. After the reverse transcription step, barcoded cDNA was purified with Dynabeads, and PCR amplification was performed as follows. A 5′ gene expression library was constructed on the basis of amplified cDNA. For gene expression library construction, 50 ng of amplified cDNA was fragmented and end-repaired, 150-bp paired-end reads and raw BCL files were produced after the cDNA was double size-selected with SPRIselect beads and sequenced on a NovaSeq platform (Illumina). Raw data in FASTQ format were assembled from the raw BCL files using Illumina’s bcl2fastq converter.

### ScRNA-seq data processing and sample aggregation

We used Cell Ranger Software Suite (v.3.1.0) to manipulate the FASTQ files, aligned the sequencing reads to the GRCh38 reference transcriptome build using STAR [15] and generated a filtered UMI expression profile for each single cell. Cell barcodes were then automatically determined based on the distribution of UMI counts. The following inclusion criteria were then applied to each cell: gene number between 500 and 6000, UMI count> 1000 and mitochondrial gene percentage < 10%. Genes expressed in less than three cells were excluded. DoubletDecon was used to detect and remove doublets. After filtration, a total of 13,526 cells were retained for the following analysis. Finally, “FindIntegrationAnchors” and “IntegrateData” in Seurat v.3 were used to integrate the filtered gene-barcode matrix of all samples to remove batch effects across different donors [16]. “ccRemover” was used to identify and remove cell-cycle effect [17].

### Cell clustering and annotation

“LogNormalData” in Seurat v.3 was used to normalize the filtered gene-barcode matrix. Then, the top 2000 highly variable genes were calculated with the “Find Variable Features” function in Seurat. Principal component analysis (PCA) was conducted by using the top 2000 variable genes. Then, the top 50 principal components were used to perform UMAP to visualize the cells. In addition, cell clustering was carried out on the basis of PCA-reduced data for clustering analysis with Seurat v.3. Cell clusters were annotated according to SingleR [18] and marker genes; for example, the phenotypes *CD3E*+ *CD4*+ , *CD3E*+ *CD8A*+ and *CD79A*+ were used to identify CD4+ T, CD8+ T and B cells, respectively.

### *Reclustering of CD4*+ *T cells, CD8*+ *T cells, B cells and myeloid immunocytes*

CD4+ T cells, CD8+ T cells, B cells and myeloid immunocytes annotated according to SingleR and marker genes were reintegrated and reclustered by Seurat v.3. Reclustered CD4+ T cells, CD8+ T cells, B cells and myeloid immunocyte subclusters were named by cluster number and cell type; for example, cluster 3 in CD8+ T cells was named C3–CD8. Specifically. With the help of canonical correlation analysis and PCA of the first 50 dimensions, CD4+ T cells, CD8+ T cells, B cells and myeloid immunocytes of all samples were integrated. The parameter k.filter was set to 120. In the clustering step, the parameter resolution was set to 0.8. The “Monocle 3” in Seurat ordered CD8+ T single cells in an unsupervised manner and was used to analyze single-cell trajectories along pseudotime to discover the developmental transitions of CD8+ T subclusters.

### GO enrichment analysis

GO enrichment analysis of differentially expressed gene sets was carried out in the GOseq R and KOBAS 3.0 packages. GO terms with adjusted P values below 0.05 were considered significantly enriched among differentially expressed genes.

### Cellular interaction analysis

CellPhoneDB (v2.1.7) [19] was used to classify cellular interactions for cells derived from ECCA tumor and matched paratumor tissues. Receptor–ligand crosstalk between two cell populations was identified according to the expression of receptors of one cell type and ligands of the other cell type. The expression frequency of a given receptor ligand gene was required to be > 10% in the corresponding cluster. The* P* value indicated the likelihood of a given receptor–ligand complex by calculating the proportion of means equal to or higher than the actual mean. The significant mean shows the total mean of the average expression values of each partner in the corresponding cell type interaction pair.

## Results

### Immunocyte clustering and analysis

**Fig. 1:**
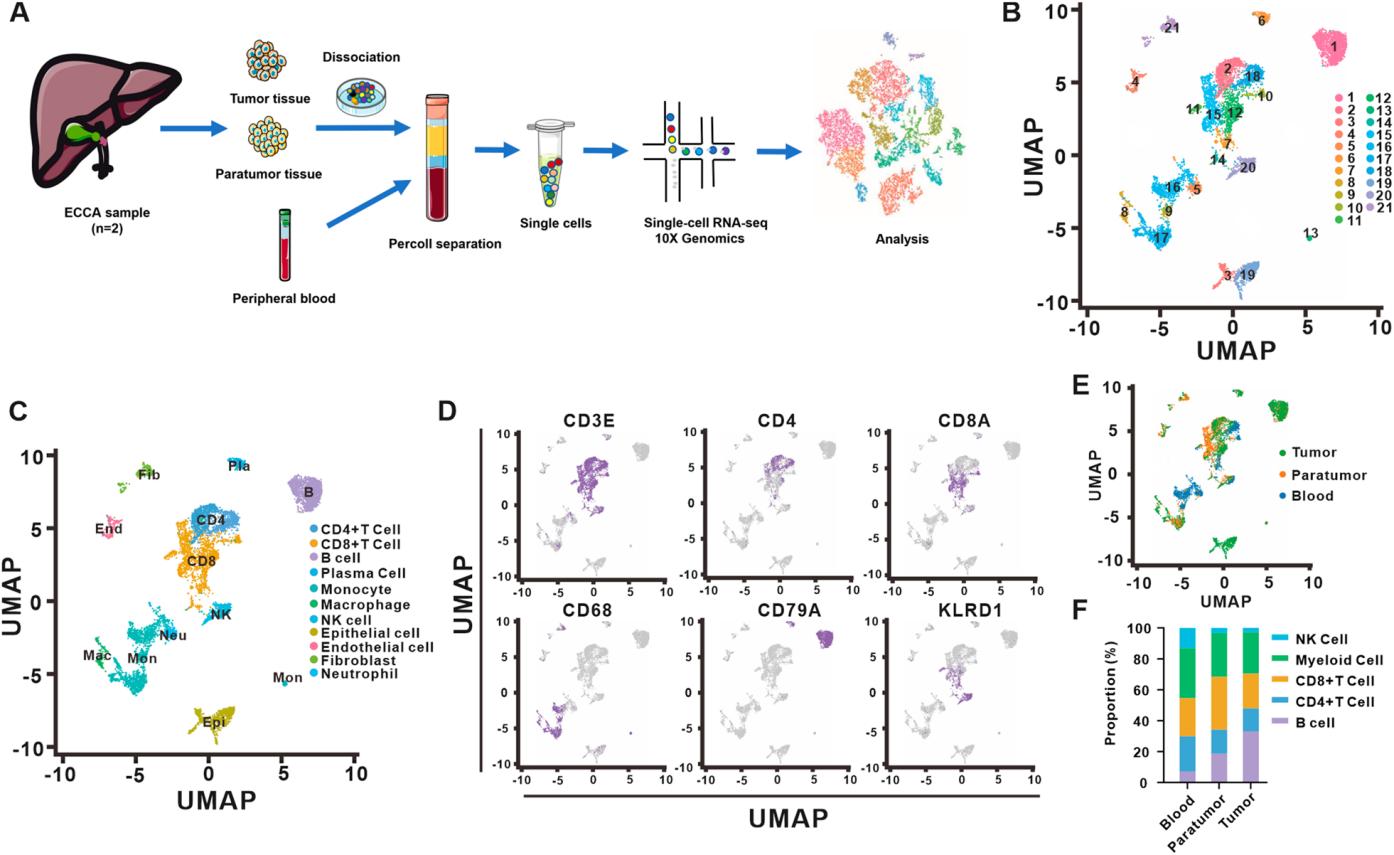
Immune cell heterogeneity in ECCA. **A** Overview of the workflow in our study. **B** UMAP plot of the cells from ECCA patients, with each cell color-coded by cell cluster according to gene expression characteristics. **C** UMAP plot of the cell clusters annotated by the SingleR package. **D** UMAP plot of the cell clusters annotated by the marker genes *CD3E*, *CD4*, *CD8A*, *CD68*, *CD79A* and *KLRD1*. **E** UMAP plot of color-coded cells from the tumor tissues, paratumor tissues and peripheral blood of ECCA patients. **F** The proportions of B cells, CD4 + T cells, CD8 + T cells, myeloid immune cells and NK cells in peripheral blood, paratumor, and tumor tissues from ECCA patients

To explore the cellular heterogeneity of immunocytes in ECCA, we analyzed the immunocytes from ECCA patients based on scRNA-seq (Fig. 1A). RNA-seq data derived from a total of 13,526 sorted single cells were divided into 21 clusters and named cluster 1–21 (Fig. 1B). Significantly differentially expressed genes in each cluster are shown in Additional file 4: Table S2. According to the SingleR package [20] and marker genes, subclusters 7, 10, 11, 12, 14 and 15 were identified as CD8+ T lymphocytes with marker genes *CD3E* and *CD8A*, subclusters 2 and 18 were CD4+ T lymphocytes with marker gene *CD3E* and *CD4*, clusters 1 and 6 were B lymphocytes with *CD79A*, subcluster 20 was NK cells with *KLRD1*+ , and clusters 8, 9, 13, 16 and 17 were monocytes/macrophages with *CD68* (Fig. 1C, D). Cluster 5 (neutrophils, *FCGR3B*+), cluster 4 (endothelial cells, *PLVAP*+), clusters 3 and 19 (epithelial cells, *KRT18*+) and cluster 21 (fibroblasts, *C1S*+) were mixed nontargeted cells and excluded from the following study (Fig. 1C and Additional file 1: Fig. S1A).

Our study revealed that the proportion of B lymphocytes gradually increased in peripheral blood (6.93%), paratumor tissues (18.57%) and tumor tissues (33.22%). In contrast, the proportions of CD4+ T lymphocytes gradually decreased from 22.98%, 15.59% 3.31% in peripheral blood, paratumor tissues and tumor tissues, respectively, which was also observed in NK clusters at 13.12%, 3.31% and 2.69%, respectively. Notably, the proportion of CD8+ T lymphocytes in tumor tissues (22.47%) was less than that in paratumor tissues (34.16%) (Fig. 1E, F).

### *CD4*+ *T lymphocyte reclustering and subcluster analysis*

**Fig. 2:**
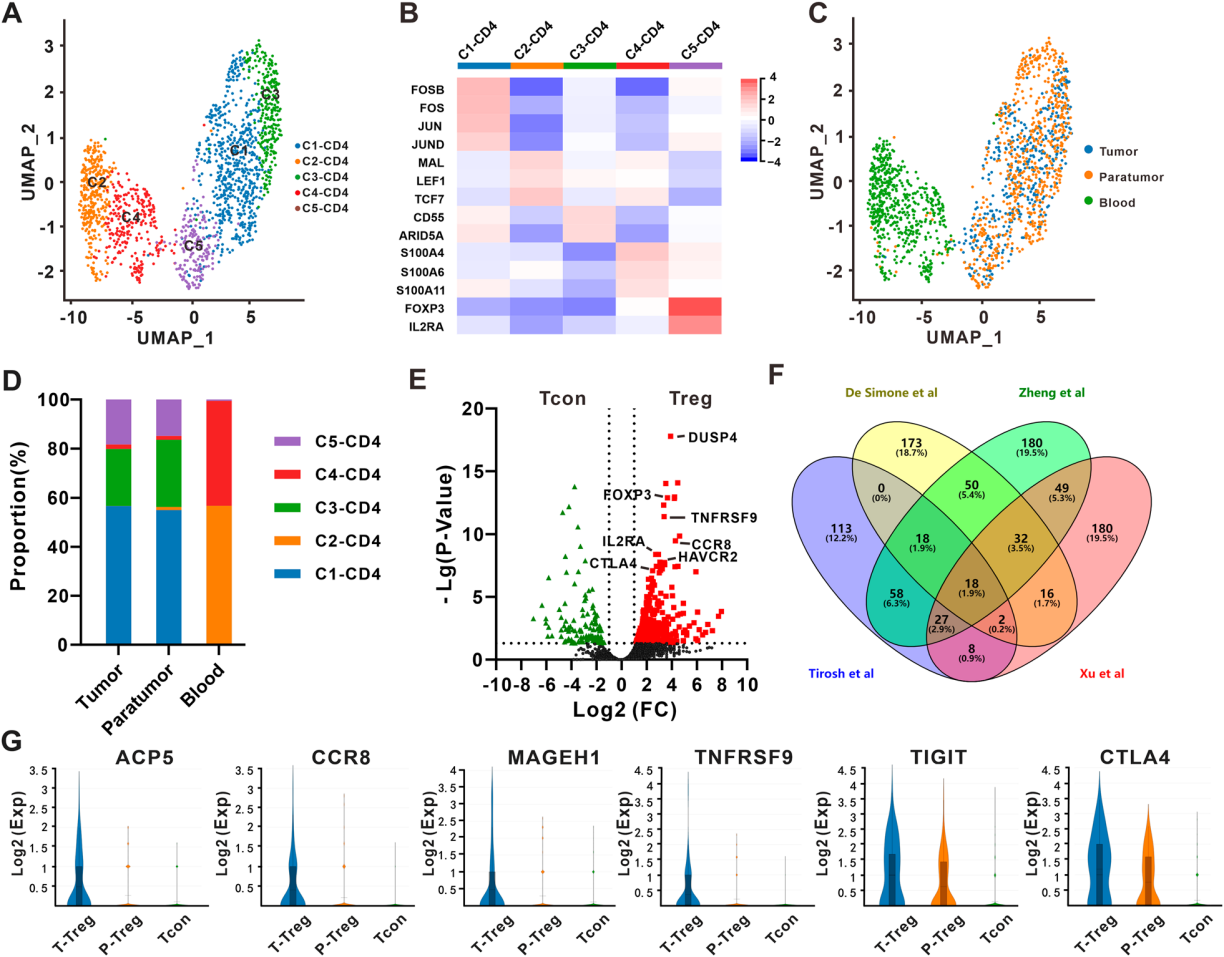
CD4 + T cell heterogeneity in ECCA. **A** UMAP plot of CD4 + T cells from ECCA patients, with each cell color coded by cell subcluster according to gene expression characteristics. **B **The heatmap shows the normalized mean expression of marker genes in each CD4 + T cell subcluster. **C** UMAP plot of the color-coded CD4 + T cells from tumor tissues, paratumor tissues and peripheral blood from ECCA patients. **D** The proportion of five CD4 + T cell subclusters in peripheral blood, paratumor, and tumor tissues from ECCA patients. **E** Volcano plot showing differentially expressed genes between conventional T cells (Tcons) and Treg cells. Each red square denotes a highly expressed gene in Treg cells that met the p value and fold difference criteria. **F** Venn diagram showing the overlap of Treg genes identified in this study with those from previous studies by Zheng et al. [10], De Simone et al., [27] and Tirosh et al. [13] **G** Violin plots showing the expression of overlapping genes recurrently identified in ECCA data and previous studies across paratumor-infiltrating Tregs (P-Tregs), tumor-infiltrating Tregs (T-Tregs) and Tcon

We reclustered the 1657 CD4+ T lymphocytes annotated with the SingleR package and marker genes (Fig. 1C, D), and 5 subclusters emerged and were named C1–CD4 to C5–CD4 (Fig. 2A). Significantly differentially expressed genes in each subcluster are shown in Additional file 5: Table S3. C1–CD4 was characterized by high expression of *FOS*, *FOSB*, *FOSL2*, *JUN* and *JUND* known as transcription factor subunits of AP-1 (Fig. 2B). These genes have been described in several satellite cell studies and have been proved to be high expressed genes induced by tissue dissociation [21]. Thus, C1–CD4 may be subcluster resulted from tissue dissociation procedure. C2–CD4 was defined as naïve T lymphocytes specifically expressing the naïve marker genes *LEF1*, *TCF7* and *MAL* (Fig. 2B) [22]. C3–CD4 highly expressed *CD55* and *ARID5A*, which are known as markers of activated T lymphocytes (Fig. 2B) [23, 24]. C4–CD4 highly expressed genes *S100A4*, *S100A6* and *S100A11*, and *S100A4* was exclusively expressed by memory CD4+ T lymphocytes (Fig. 2B) [25]. The marker genes *FOXP3* and *IL2RA* (CD25) were exclusively prevalent in C5–CD4, which was finally identified as regulatory CD4+ T lymphocytes (CD4+ Tregs) (Fig. 2B). An obvious distinct tissue distribution was found in the above subclusters. The resting CD4+ T lymphocyte subclusters (C2–CD4 and C4–CD4) contained mostly cells from peripheral blood, while activated CD4+ T lymphocyte subclusters (C3–CD4 and C5–CD4) were almost exclusively populated in tumor tissues and paratumor tissues (Fig. 2C, D).

Tregs are characterized as inhibitors of antitumor immunity and promote tumor development and progression. It is of great importance to see a strong enrichment of Tregs (C5–CD4) in ECCA tumor and paratumor tissues in our study (Fig. 2C, D). Furthermore, compared with conventional T cells (Tcon, from C1–CD4 to C4–CD4), Tregs (C5–CD4) exclusively expressed 333 genes in this study, including marker genes (*FOXP3* and *IL2RA*), costimulatory receptors (*ICOS*, *TNFRSF4*, *TNFRSF9* and *TNFRSF18*) and coinhibitory receptor/immune checkpoint genes (*CTLA4*, *TIGIT*, *HAVCR2* and *PDCD1*) (Fig. 2E and Additional file 5: Table S3). Compared to previous studies, overlapping analysis showed 18 Treg-specific genes, including *IL2RA, FOXP3, CCR8, TNFRSF18, BATF, TNFRSF4, CTSC, IL2RB, CTLA4, ACP5, TIGIT, ICOS, IL1R2, TNFRSF9, NCF4, IKZF2, VDR* and *MAGEH1* (Fig. 2F and Additional file 6: Table S4) [22, 26, 27]. Among them, *CTLA4, TIGIT, TNFRSF4* and *TNFRSF18* were highly expressed in tumor-infiltrating Tregs. Moreover, *ACP5, MAGEH1, TNFRSF9* and *CCR8* were especially populated in tumor-infiltrating Tregs and *IKZF2* was only highly expressed in paratumor-infiltrating Tregs (Fig. 2G and Additional file 1: Fig. S1B).

### *CD8*+ *T lymphocyte reclustering and subcluster analysis*

**Fig. 3:**
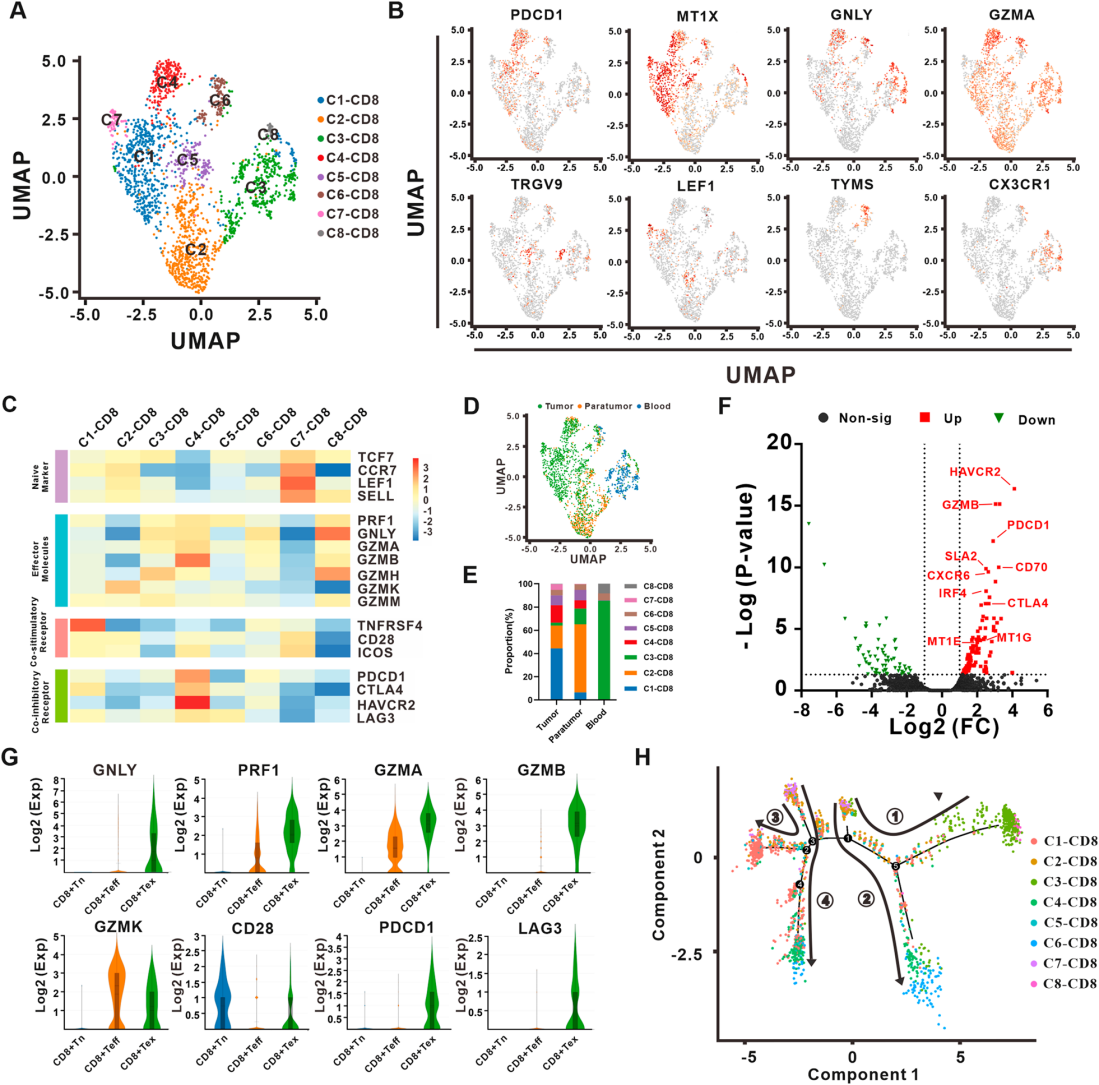
CD8 + T cell heterogeneity in ECCA. **A** UMAP plot of CD8 + T cells from ECCA patients, with each cell color coded by cell subcluster according to gene expression characteristics. **B** UMAP plot of the CD8+ T cell subclusters annotated by marker genes. **C** The heatmap shows the normalized mean expression of differentially expressed genes (rows) associated with the selected CD8 + T cell functions in each CD8 + T cell subcluster. **D** UMAP plot of color-coded CD8 + T cells from the tumor tissues, paratumor tissues and peripheral blood of ECCA patients. **E** The proportion of eight CD8 + T cell subclusters in peripheral blood, paratumor, and tumor tissues from ECCA patients. **F** Volcano plot showing differentially expressed genes in CD8 + Tex cells. Each red square denotes a highly expressed gene that met the p value and fold difference criteria. **G** Violin plots showing the normalized mean expression of differentially expressed genes associated with the selected CD8 + T cell functions across CD8 + Tn (C7-CD8), CD8 + Teff (C2-CD8, C3-CD8 and C8-CD8) and CD8 + Tex (C1-CD8 and C4-CD8) cells. **H** The ordering of CD8 + T cells along pseudotime in a two-dimensional state space defined by Monocle2. Each point corresponds to a single cell, and each color represents a CD8 + T cell subcluster

To illustrate the CD8+ T subclusters in ECCA, we reclustered 1657 cells identified as CD8+ T lymphocytes and obtained 8 subclusters named C1–CD8 to C8–CD8 (Fig. 3A). Significantly differentially expressed genes in each subcluster are shown in Additional file 7: Table S5. CD8+ Tex subclusters C1–CD8 and C4–CD8 were characterized by high expression of the T cell exhaustion marker genes *PDCD1*, *CTLA4*, *LAG3* and *HAVCR2* and the metallothionein family genes *MT1E*, *MT1F* and *MT1X* (Fig. 3B and Additional file 1: Fig. S1C) [28]. Subclusters C2–CD8, C3–CD8 and C8–CD8, described as effector CD8+ T lymphocytes (CD8+ Teff), presented a strong degree of expression of the cytotoxic effectors *PRF1*, *GNLY* and *GZM* and low expression of exhaustion markers (Fig. 3B, C and Additional file 1: Fig. S1C). The γδ-TCR CD8+ T lymphocyte subclusters C5–CD8 were characterized by high expression of the TCR γ and δ subunit genes *TRDV2*, *TRGV9*, *TRGV3* and *TRGV2* (Fig. 3B). Additionally, the naïve markers *SELL*, *CCR7*, *LEF1* and *TCF7* and the costimulatory receptors *ICOS* and *CD28* were enriched in subcluster C7–CD8 T cells and identified as naïve CD8+ T lymphocytes (Fig. 3B, C and Additional file 1: Fig. S1C). C6–CD8 cells specifically expressed *TYMS* and *PCLAF* associated with DNA replication and repair, suggesting a proliferative CD8+ T lymphocyte subcluster (Fig. 3B, C and Additional file 1: Fig. S1C). Moreover, in the analysis of CD8+ T lymphocyte subcluster distribution, we found that CD8+ Tex subclusters C1–CD8 and C4–CD8 showed great preference for tumor tissue (Fig. 3D, E). A previous study proved that CX3CR1+ CD8+ T cells exhibited robust cytotoxicity [29]. Interestingly, CD8+ Teff subclusters C3–CD8 and C8–CD8 highly expressed *CX3CR1* were enriched in peripheral blood, while C2–CD8 weakly expressed CX3CR1 concentrated in paratumor tissues (Fig. 3D, E).

CD8+ Tex lymphocytes feature loss of functional effect, upregulation of multiple inhibitory receptor expression and inability to convert to memory CD8+ T lymphocytes to participate in the effective immune response of the body upon receipt of a new stimulus. This subcluster plays an extremely critical role in the TME. Consistent with previous studies, CD8+ Tex lymphocytes in ECCA presented preferential enrichment in tumor tissue with strong expression of coinhibitory receptors. Compared with CD8+ Tn (C7–CD8) and CD8+ Teff (C2–CD8, C3–CD8 and C8–CD8), CD8+ Tex (C1–CD8 and C4–CD8) highly expressed 110 genes (Fig. 3F). Notably, the average expression of the cytotoxic effectors *PRF1*, *GNLY*, *GZMA* and *GZMB* in CD8+ Tex cells was even higher than that in CD8+ Teff cells (Fig. 3G). Compared with C4–CD8, CD8+ Tex subcluster C1–CD8 highly expressed the costimulatory receptor *TNFRSF4* but expressed low levels of the coinhibitory receptors *PDCD1*, *CTLA4*, *HAVCR2* and *LAG3* (Fig. 3C). In addition, pseudotime trajectory analysis confirmed the differentiation trajectory of these subclusters, successively, from Tn (C7–CD8) to αβ-TCR CD8+ Teff (C2–CD8) or γδ-TCR CD8+ Teff (C5–CD8) to CD8+ Tex (C1–CD8) then to Tex (C4–CD8) or proliferative subcluster (C6–CD8) (Fig. 3H). Thus, it is reasonable to identify that the C1–CD8 may be the "intermediate" CD8+ Tex between CD8+ Tn (C7–CD8) and "complete" CD8+ Tex (C4-CD8) (Fig. 3H). A total of 124 genes were highly expressed by "intermediate" CD8+ Tex subcluster C1–CD8 and GO enrichment analysis revealed that these genes were enriched in the pathways of protein folding and binding and response to heat and zinc ions (Additional file 2: Fig. S2A). A total of 266 genes were highly expressed by the "complete" CD8+ Tex subcluster C4–CD8 and GO enrichment analysis revealed that these genes were enriched in the pathways of T cell aggregation, adhesion and activation (Additional file 2: Fig. S2B).

### B lymphocyte reclustering and subcluster analysis

**Fig. 4:**
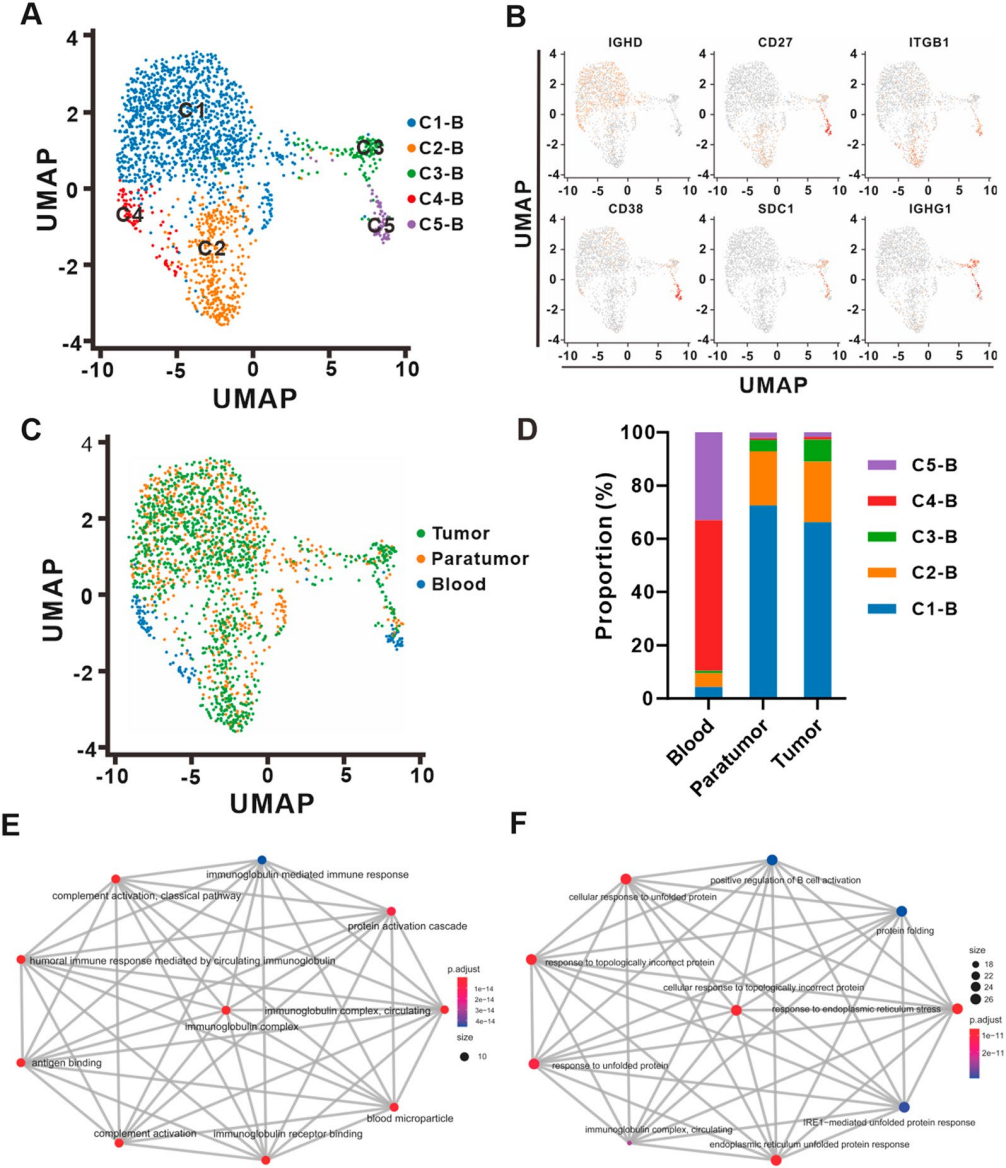
B cell heterogeneity in ECCA. **A** UMAP plot of the B cells from ECCA patients, with each cell color-coded by cell subcluster according to gene expression characteristics. **B** UMAP plot of the B cell subclusters annotated by marker genes. **C** UMAP plot of color-coded B cells from the tumor tissues, paratumor tissues and peripheral blood of ECCA patients. **D** The proportion of five B cell subclusters in peripheral blood, paratumor, and tumor tissues from ECCA patients. **E** GO enrichment analysis of the nonclassic PC subcluster C3-B showing enriched pathways. **F** GO enrichment analysis of the classic PC subcluster C5-B showing enriched pathways

A total of 2038 B lymphocytes were reclustered, and 5 subclusters named C1–B to C5–B lymphocytes were mapped for further study (Fig. 4A). Significantly differentially expressed genes in each subcluster are shown in Additional file 8: Table S6. C1–B and C4–B were classified as naïve B cells (Bn, *CD27-IgD*+) with high expression of the naïve marker genes *IL4R*, *IGHD (IgD)* and *IGHM* (*IgM*) (Fig. 4B and Additional file 2: Fig S2C). C2–B showed high expression of the marker gene *CD27* and the genes *ITGB1* and *S100A6*, which indicated memory B cells (Bm, *CD27*+ *IgD−*) (Fig. 4B and Additional file 2: Fig S2C) [30]. Additionally, C3–B and C5–B subclusters were plasmacytes (PCs) depending on the high expression of the marker genes *CD27* and *SDC1* (*CD138)* and the genes *IGHG1*, *IGHG4*, *MZB1* and *XBP1* (Fig. 4B and Additional file 2: Fig. S2C). The classic PCs C5–B (*CD27*+ *CD138*+ *CD38*+) were characterized by high co-expression of the marker genes *CD138* and *CD38*, while C3–B (*CD27*+ *CD138*+ *CD38−*) showed quite poor levels of *CD38*, indicating a “nonclassic” PC subcluster rarely described in previous studies (Fig. 4B). Compared with peripheral blood, tumor and paratumor tissues contained higher proportions of Bn subcluster C1–B and Bm subcluster C2–B; conversely, the Bn subcluster C4–B and PC subcluster C5–B were observed infrequently. The proportion of “nonclassic” PC subcluster C3–B increased gradually in peripheral blood (0.87%), paratumor tissues (4.27%) and tumor tissues (8.30%) (Fig. 4C, D).

Sixty-three genes were highly expressed in nonclassic PC subcluster C3–B, and *IGHG4*, *IGHG1*, *MZB1*, *SDC1*, *DERL3*, *XBP1*, *PRDX4*, *FKBP11*, *SSR4* and *IGLV3*-*1* were the top 10 highly expressed genes. GO enrichment analysis revealed that highly expressed genes were enriched in the pathways of immunoglobulin complex and complement activation (Fig. 4E). Analysis of C5–B showed a total of 431 highly expressed genes, and *TRIB1*, *AQP3*, *B9D1*, *CYTOR*, *CNKSR1*, *FNDC3B*, *IGF1*, *TNFRSF17*, *TXNDC5* and *SLAMF7* were the top 10 highly expressed genes. GO enrichment analysis revealed that highly expressed genes were enriched in the immunoglobulin complex and response to protein folding (Fig. 4F).

### Myeloid immunocyte reclustering and subcluster analysis

**Fig. 5:**
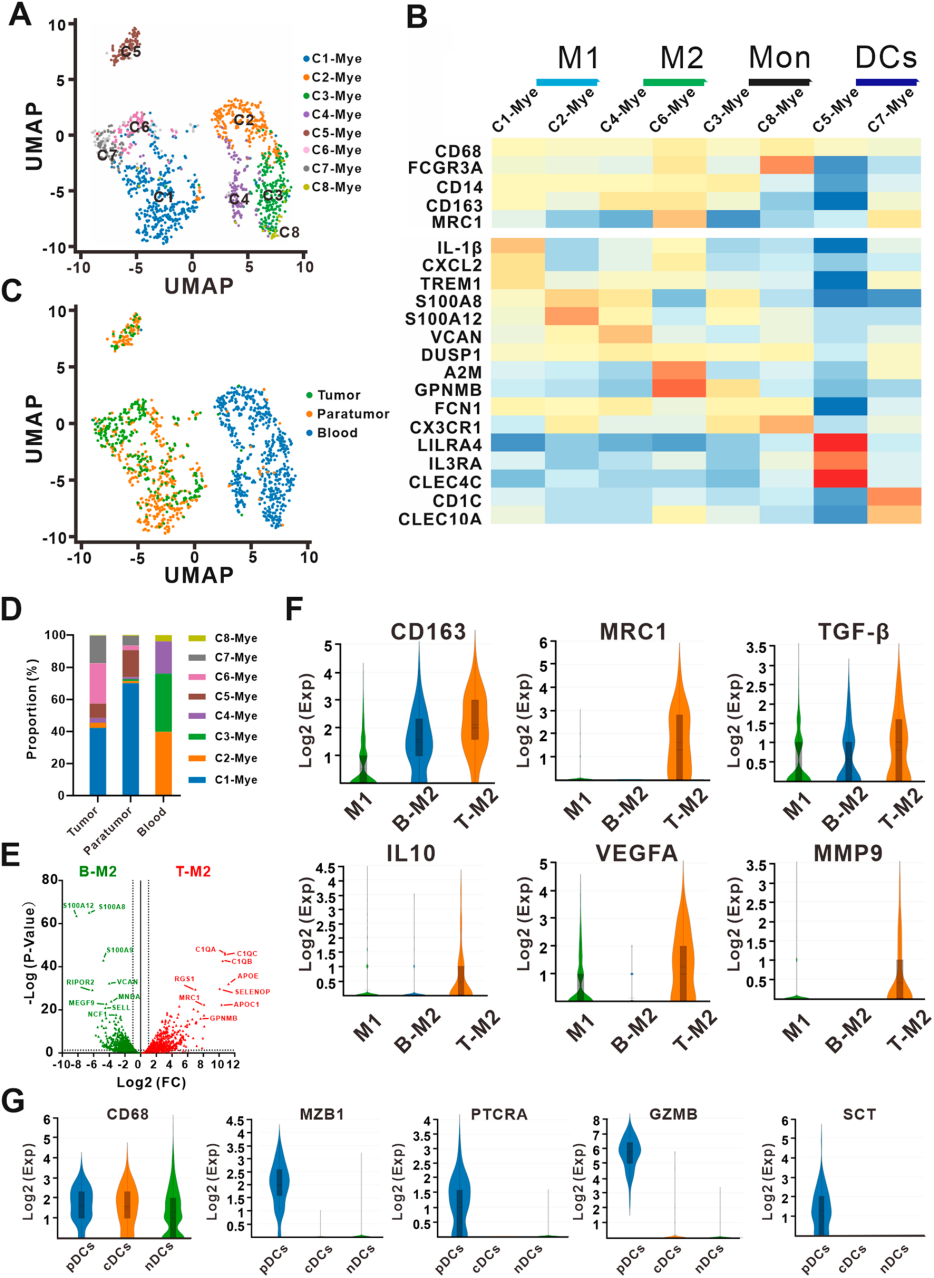
Myeloid immune cell heterogeneity in ECCA. **A** UMAP plot of myeloid immune cells from ECCA patients, with each cell color coded by cell subcluster according to gene expression characteristics. **B** The heatmap shows the normalized mean expression of marker genes in each myeloid immune cell subcluster. **C** UMAP plot of color-coded myeloid immune cells from the tumor tissues, paratumor tissues and peripheral blood of ECCA patients. **D** The proportions of eight myeloid immune cell subclusters in peripheral blood, paratumor, and tumor tissues from ECCA patients. **E** Volcano plot showing differentially expressed genes between blood M2 macrophages (C4-Mye) and tumor and paratumor M2 macrophages (C6-Mye). Each green and red triangle denotes a highly expressed gene in blood (B-M2, C4-Mye) and tumor/paratumor (T-M2, C6-Mye) M2 macrophages that met the p value and fold difference criteria, respectively. **F** Violin plots showing the normalized mean expression of M2 macrophage-related marker genes and protumorigenic genes across M1 macrophages (M1, C1-Mye and C2-Mye), blood M2 macrophages (B-M2, C4-Mye) and tumor and paratumor M2 macrophages (T-M2, C6-Mye). **G** Violin plots showing the normalized mean expression of characteristic genes of pDCs across pDCs (C5-Mye), cDCs (C7-Mye) and non-DCs (nDCs, other myeloid immune cell subclusters except DCs)

To characterize myeloid immune cells in ECCA, we reclustered 1657 myeloid immunocytes and identified 8 subclusters named C1-Mye to C8-Mye (Fig. 5A). Significantly differentially expressed genes in each subcluster are shown in Additional file 9: Table S7. C1-Mye highly expressed *IL*-*1β*, *CXCL2*, *SOD2*, *TREM1* and *AREG*, and C2-Mye highly expressed *S100A8*, *S100A12* and *LYZ* (Fig. 5B). Both of the subclusters above had gene expression profiles suggesting M1 macrophages [31–33]. As C4-Mye highly expressed M2-specific genes *VCAN* and *DUSP1* and marker genes *CD163* and C6-Mye highly expressed M2-specific genes *TGF*-*β1*, *A2M*, *MAF*, *SLC40A1*, *FOLR2*, *GPNMB* and *MSR1* and the marker genes *CD163* and *MRC1* (*CD206*), C4-Mye and C6-Mye were defined as M2 macrophages (Fig. 5B). C3-Mye was classified as CD14+ classical monocytes with high expression of *CD14* and *FCN1* but low expression of *FCGR3A* (*CD16*), and C8-Mye highly expressed *FCGR3A* (*CD16*), *CX3CR1* and *FCN1* but expressed low levels of *CD14*, indicating CD16+ nonclassic monocytes (Fig. 5B). C5-Mye with high expression of the marker genes *LILRA4*, *IL3RA* and *CLEC4C* resembled plasmacytoid dendritic cells (pDCs), and classical dendritic cells (cDCs) pointed to C7-Mye because of their high expression of the marker genes *CD1C* and *CLEC10A* (Fig. 5B) [34, 35]. Furthermore, dendritic cell subclusters C5-Mye and C7-Mye were enriched in tumor and paratumor tissues, while monocyte subclusters C3-Mye and C8-Mye were concentrated in peripheral blood (Fig. 5C, D). The macrophage subclusters C1-Mye (M1) and C6-Mye (M2) were almost exclusively populated with cells from tumor/paratumor tissues, while C2-Mye (M1) and C4-Mye (M2) were almost exclusively populated with cells from peripheral blood (Fig. 5C, D). These different distributions of macrophage subclusters indicated separate transcriptome characteristics of M1 and M2 macrophages between tissues/paratumor tissues and peripheral blood. In addition, the proportion of C1-Mye (M1) in paratumor tissues (70.09%) was higher than that in tumor tissues (42.18%), while the proportion of C6-Mye (M2) in paratumor tissues (2.78%) was lower than that in tumor tissues (25.09%) (Fig. 5D).

**Fig. 6:**
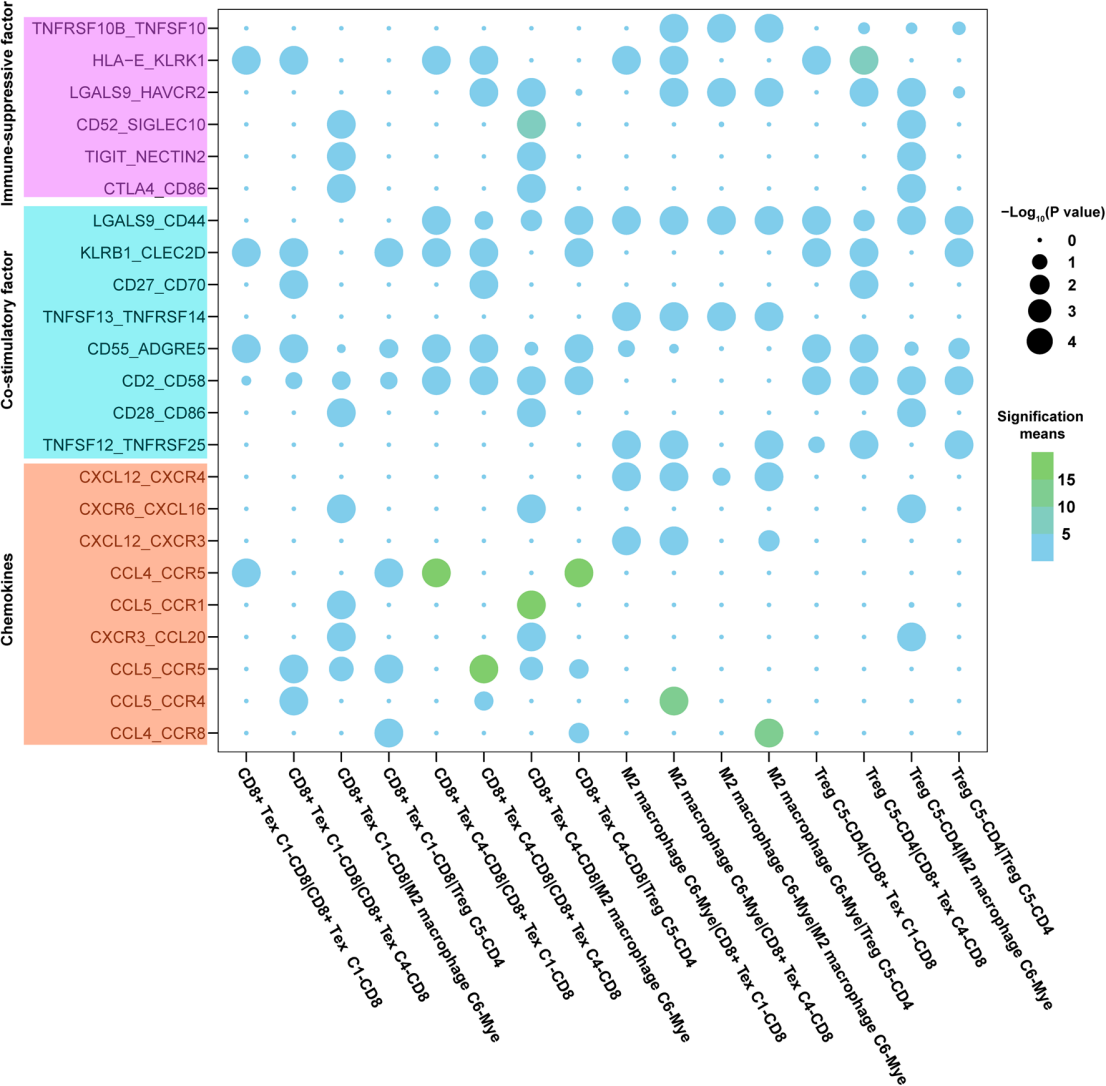
Intercellular cross-talk between immune cells in ECCA. Dot plots present selected ligand–receptor cross-linkages between two immune clusters for Tregs, the exhausted CD8 + T lymphocytes C1-CD8/C4-CD8 and the M2 macrophages C6-Mye. The significant means of the expression levels of two interacting molecules are shown by a color heatmap (right panel), with light blue to green representing low to high expression. The P values from a one-sided permutation test are shown by circle size. Different colors of squares on the left side indicate different functional factors involved in receptor–ligand crosstalk

As they showed potent protumorigenic and immunosuppressive properties and were enriched in tumor tissues, the M2 macrophage subclusters C4-Mye and C6-Mye were further analyzed. In contrast to C4-Mye enriched in blood, C6-Mye preferred tumor and paratumor tissue and highly expressed 591 genes (Fig. 5E), including the M2 macrophage marker gene *MCR1* (*CD206*), and highly expressed the M2 macrophage-related protumorigenic genes *TGF*-*β*, *IL10*, *VEGFA and MMP9* (Fig. 5F). Although studies have focused on pDC differentiation within the myeloid lineage, pDCs in our study showed transcriptome characteristics of both lymphoid and myeloid lineages. The marker genes *CD68* (monocytes/macrophages), *MZB1* (plasmacytes), *PTCRA* (immature T lymphocytes) and *GZMB* (NK cells and cytotoxic T lymphocytes) were the top-ranked genes coexpressed by pDCs (Fig. 5G). pDCs were suggested to serve an endocrine function because highly expressed *SCT* (secretin) may have endocrine functions (Fig. 5G).

### Intercellular cooperation network of immunocytes in ECCA

To elucidate cell-to-cell communication in ECCA, CellPhoneDB software was applied to identify potential ligand–receptor interactions in various subclusters originating from ECCA tumor and paratumor tissues (Additional file 10: Table S8). Based on the cell interaction network counts (Additional file 11: Table S9), we found intense cell-to-cell interactions among M2 macrophages (C6-Mye), Tregs (C5–CD4) and CD8+ Tex cells (C1–CD8 and C4–CD8) via chemotactic, immune-suppressive or costimulatory factors (Fig. 6). Among these cells, Tregs highly expressed the *TIGIT*, *CTLA4* and *CCR8* genes, which showed receptor–ligand interactions with *NECTIN2*, *CD86* and *CCL4* from M2 macrophages (C6-Mye), implying potential cross-talk between M2 macrophages (C6-Mye) and Tregs (C5–CD4). In addition, Treg cells (C5–CD4) were suggested to interact intensively with CD8+ Tex cells (C1–CD8 and C4–CD8) through *HLA*-*E*-*KLRK1* and its subtypes, which were classified as immune inhibitors because they promote senescent cell accumulation [36] and suppress tumor-specific cytotoxic T lymphocytes [37], and *HLA*-*E*-*KLRK1* was identified as a novel checkpoint in the tumor microenvironment (TME) [38]. In addition, Treg cells (C5–CD4) were suggested to communicate with CD8+ Tex cells via chemotactic factors (*CCL4*–*CCR8* and *CCL5*–*CCR5*) [39, 40], adhesion molecules (*ICAM3*-*aLb2* complex) and immune regulatory molecules (*LGALS9*-*CD44*) [41], which were abundant in the TME and upregulated the function of Tregs and CD8+ Tex cells. M2 macrophages (C6-Mye) were predicted to secrete the factors *CXCL12* and *CCL4* to attract CD8+ Tex cells by binding to *CXCR3/CXCR4* and *CCR5* on CD8+ Tex cells, which are known to recruit immunocytes into cancer tissues. Notably, M2 macrophages were observed to develop self-amplifying feedback via the *CXCL12*-*CXCR4* axis, which was shown to induce tumor-promoting M2 polarization of macrophages [42, 43]. *NRP1/2*-*VEGFA* was predicted to interact within M2 macrophages, which could potentially contribute to tumor angiogenesis [44].

## Discussion

It is widely known that CCA has a poor prognosis and is a challenging malignancy characterized by late diagnosis, low resectability, early recurrence, drug resistance and especially poor prognosis. Immunocytes exhibit obvious heterogeneity and promote or suppress cancer progression. Previous studies have identified immunotherapeutic targets, including PD1, PDL1 and CTLA4, that promote the treatment of non-small-cell lung cancer, melanoma and renal carcinoma. Deeper insight into the heterogeneity of immunocytes in CCA is urging, and it is promising to provide potential immunotherapeutic targets. Herein, based on scRNA-seq, we analyzed immunocytes of ECCA patients to describe the transcriptome characteristics and potential function of each immunocyte subcluster.

As CD4+ Treg cells promote tumor immunosuppression and are associated with poor prognosis, novel immunotherapy targets for Treg cells may improve the treatment of ECCA. Our study analyzed Treg cells and revealed 333 exclusively expressed genes. Eighteen genes identified in our study were consistent with those reported by earlier studies in melanoma, breast, colon, lung and liver cancers, including coinhibitory receptors *CTLA4* and *TIGIT* and costimulatory receptors *TNFRSF4* and *TNFRSF18*. Previous studies have proven that CTLA4, TIGIT, TNFRSF4 and TNFRSF18 cannot promote and instead suppress the proliferation and immunosuppression of Tregs [45–47]. Among the 18 Treg-specific overlapping genes, *ACP5, MAGEH1, TNFRSF9 and CCR8* were exclusively expressed in tumor-infiltrating Tregs and are potential immunotherapy targets for tumor-infiltrating Tregs.

In our study, CD8+ Teff subclusters in peripheral blood, not in tumor/paratumor tissues, highly express *CX3CR1* which is related to strong cytotoxic effector function, unique migration pattern and positioning adjacent to the entry site of pathogens [29]. Interestingly, the expression levels of the cytotoxic effectors *PRF1*, *GNLY*, *GZMA*, *GZMB* and *GZMH* in CD8+ Tex subclusters were even richer than those in CD8+ Teff subclusters, which implied that CD8+ Tex cells still tended to respond to cancer cells. However, the CD8+ Tex subclusters, characterized by high expression of coinhibitory receptors, presented limited proliferation and differentiation, which suggested an immunosuppressive TME in ECCA. Moreover, our study revealed two CD8+ Tex subclusters (C1–CD8 and C4–CD8). Compared with subcluster C4–CD8, C1–CD8 weakly expressed coinhibitory receptors but highly expressed *MT1* which correlates with loss of effector function and acquisition of a dysfunctional phenotype of CD8+ T cells [28]. In addition, pseudotime trajectory analysis confirmed that C1–CD8 could differentiate into C4–CD8. Thus, C1–CD8 may consist of "intermediate" CD8+ Tex cells and could differentiate into "complete" CD8+ Tex cells C4–CD8.

Consensus exists the utility of combined assessment of CD38 and CD138 for the identification of plasma cells, and CD38 emerged as a highly valuable PC-identification marker and can be reliably used for the identification of both normal and myeloma PCs. In our study, in addition to the classical PC subcluster coexpressed marker genes *CD138* and *CD38*, a rarely described “nonclassic” PC subcluster with low levels of *CD38* was observed. GO enrichment analysis revealed that the “nonclassic” PC subcluster was associated with the pathways of immunoglobulin complex and complement activation, which may be related to the regulation of innate immunity.

M2 macrophages are involved in the anti-inflammatory immune response and promote tumor activity. Compared with peripheral blood M2 macrophages, tissues/paratumor tissue M2 macrophages highly expressed the marker gene *MCR1* (*CD206*) and the protumorigenic genes *TGF*-*β*, *IL10*, *VEGFA* and *MMP9*, which indicated stronger protumorigenic and immunosuppressive properties, and *MCR1* may be a better marker to filter tumor-infiltrating M2 macrophages. pDCs are myeloid immunocytes but show both lymphoid and myeloid lineage transcriptome characteristics. In addition, pDCs highly express *SCT* and may have endocrine functions. pDCs may form an unclear cluster and need to be further studied.

A potential cell-to-cell interaction network among Tregs (C5–CD4), CD8+ Tex cells (C1–CD8 and C4–CD8) and M2 macrophages (C6-Mye) in ECCA was revealed in this study, implying the formation of a suppressive immune microenvironment in tumors via potential communication among diverse immune clusters. Consistent with previous reports, *CCR8* is hyperexpressed on Tregs, and its ligand, *CCL4*, secreted by M2 macrophages, has been demonstrated to recruit Tregs to the tumor niche in other cancers [48, 49]. Moreover, connections between TIGIT-NECTIN2 and CTLA4–CD86 have been reported in previous studies, by which Tregs could regulate their own activation [50]. *HLA*-*E*-*KLRK1* was predicted to engage in cross-talk between Tregs and CD8+ Tex cells, by which CD8+ Tex cells could escape being neutral and promote tumor progression [38]. Notably, *CCL5–CCR5* and *CCL4–CCR5* were predicted to make great contributions to interactions among Tregs(C5–CD4), CD8+ Tex cells (C1–CD8 and C4–CD8) and M2 macrophages(C6-Mye), which were characterized by recruiting and promoting activity of tumor associated immune cells, including TAM, Th17 and Treg cells [51–53]. In addition, receptor–ligand binding involving *VEGF* is densely enriched in monocyte clusters, which could contribute to tumor angiogenesis. All the potential links between diverse types of immunocytes are crucial for the preservation of homeostasis and functional regulation in the TME. Immunosuppressants, such as PD-L1 and CTLA4 inhibitors, have been moved into clinical trials for ECCA treatments, but they have not met expectations [54]. Based on the receptor–ligand analysis in this study, the immunosuppressive cross-talk of *HLA*-*E*-*KLRK1* and *LGALS9*-*CD44* between Tregs and CD8+ Tex cells suggests that they may become potential immunotherapeutic targets in ECCA.

There are several limitations to the present study. First, the sample size in our study is quite small. Compared with some solid tumors well and much studied by scRNA-seq, including intrahepatic cholangiocarcinoma, hepatocellular carcinoma, breast cancer and lung cancer, the samples, particularly paratumor tissues (weigh < 0.1 g), which we can acquire from extrahepatic cholangiocarcinoma (ECCA) patients are too small to isolate enough qualified mononuclear immune cells, as the high sample quality (single cell count > 10000 and viability > 85%) was needed to perform 10X Genomics scRNA-seq. Besides, functional assays are needed to prove our results at the protein level. However, we first clustered immunocytes and revealed immunocyte heterogeneity in ECCA-matched tumor tissues, paratumor tissues and peripheral blood based on scRNA-seq. We found potential immunotherapy targets, for example, *ACP5* and *CCR8* for tumor-infiltrating Tregs and *MT1* for CD8+ Tex cells. We also described some interesting subclusters: "intermediate" CD8+ Tex cells and the “nonclassic” PC subcluster. In addition, we proved the existence of a potential cell-to-cell interaction network among Tregs, CD8+ Tex cells and M2 macrophages in ECCA. Our study may provide a new perspective and potential targets for the treatment of ECCA.

## Supplementary Information

**Additional file 1.** **Fig. S1** (A) UMAP plot of the cell clusters annotated by marker genes *C1S*, *FCGREB*, *KRT18* and *PLVAP*. (B) Violin plots showing the expression of overlapping genes recurrently identified in CCA data and previous studies across paratumor-infiltrating Tregs (P-Tregs), tumor-infiltrating Tregs (T-Tregs) and conventional T cells (Tcon). (C) UMAP plot of the CD8 + T cell subclusters annotated by some marker genes.

**Additional file 2.** **Fig. S2** (A) The GO enrichment analysis of CD8 + Teff subclusters C1-CD8 shows enriched pathways. (B) The GO enrichment analysis of CD8 + Teff subcluster C4-CD8 shows enriched pathways. (C) UMAP plot of the B cell subclusters annotated by some marker genes

**Additional file 3.** **Table S1. **The information of ECCA patients.

**Additional file 4.** **Table S2. **List of signature genes expressed in cell clusters.

**Additional file 5. Table S3**. List of signature genes expressed in CD4+T cell subclusters, Related to Figure 2.

**Additional file 6.** **Table S4. **List of signature genes expressed in CD4+ Treg cell from diffrent studies.

**Additional file 7.** **Table S5.** List of signature genes expressed in CD8+T cell subclusters, Related to Figure 3.

**Additional file 8.** **Table S6. **List of signature genes expressed in B cell subclusters, Related to Figure 4.

**Additional file 9.** **Table S7.** List of signature genes expressed in myeloid immune cell subclusters, Related to Figure 5.

**Additional file 10. Table S8.** The receptor-ligand interactions.

**Additional file 11. Table S9.** The count of receptor-ligand interactions.

## Data Availability

Data supporting the results can be found in supplemental tables. The datasets generated during and/or analysed during the current study are available from the corresponding author on reasonable request.

## Abbreviations

Bm: Memory B lymphocyte
Bn: Naïve B lymphocyte
CCA: Cholangiocarcinoma
cDCs: Classical dendritic cells
ECCA: Extrahepatic cholangiocarcinoma
GEM: Gel bead-in-emulsion
PC: Plasmacyte
PCA: Principal component analysis
pDCs: Plasmacytoid dendritic cells
scRNA-seq: Single-cell RNA sequencing
Teff: Effector T lymphocyte
Tex: Exhausted T lymphocyte
TME: Tumor microenvironment
Tn: Naïve T lymphocyte
Treg: Regulatory T lymphocyte
