# Supplementary figures and images for: Single-cell landscape of immunocytes in patients with extrahepatic cholangiocarcinoma
Source: J Transl Med. 2022 May 13;20:210. doi: 10.1186/s12967-022-03424-5 (PMC9103331; doi:10.1186/s12967-022-03424-5)

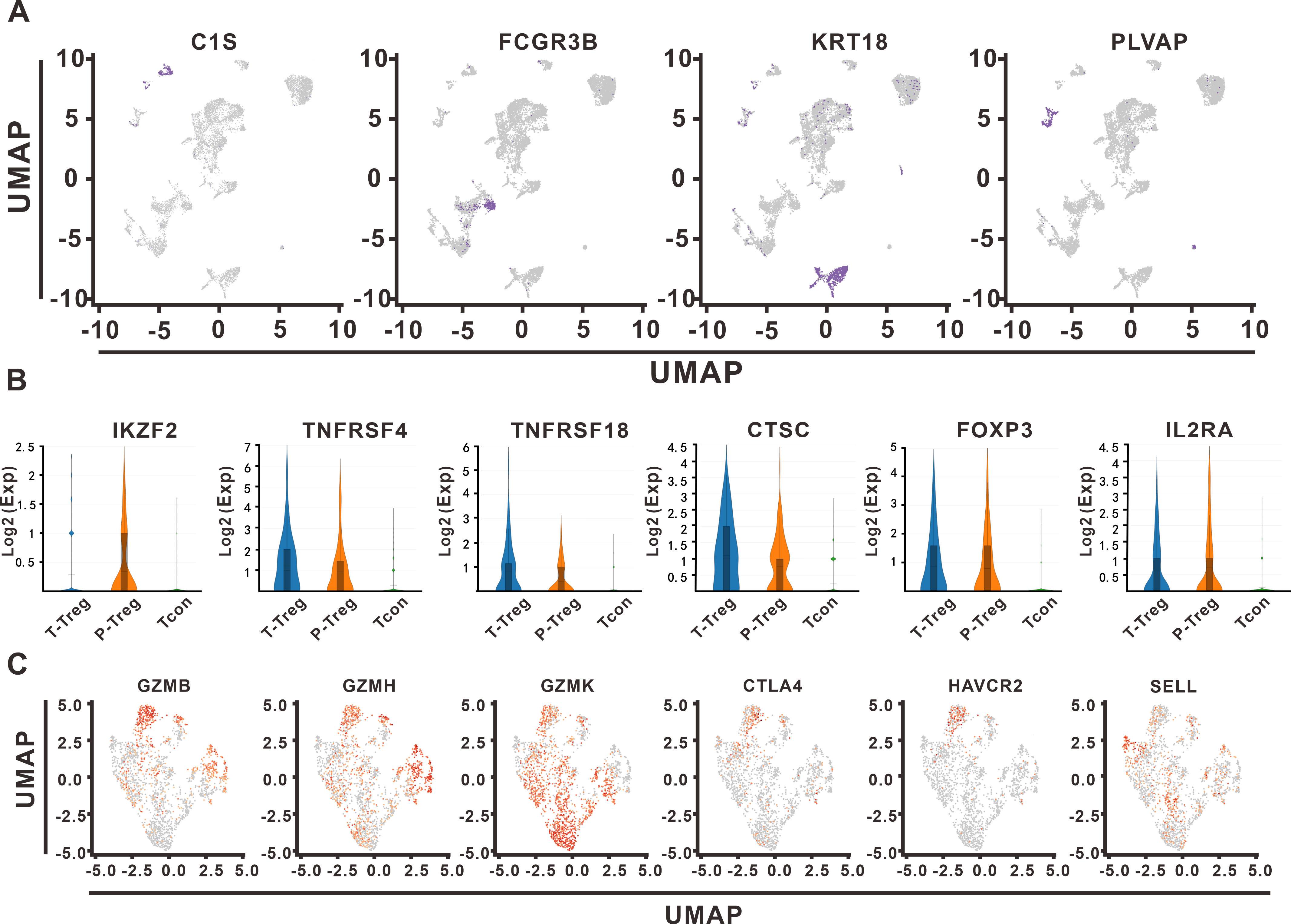

Supplement: Supplementary file 1 — Additional file 1. Fig. S1 (A) UMAP plot of the cell clusters annotated by marker genes C1S, FCGREB, KRT18 and PLVAP. (B) Violin plots showing the expression of overlapping genes recurrently identified in CCA data and previous studies across paratumor-infiltrating Tregs (P-Tregs), tumor-infiltrating Tregs (T-Tregs) and conventional T cells (Tcon). (C) UMAP plot of the CD8 + T cell subclusters annotated by some marker genes. [file 12967_2022_3424_MOESM1_ESM.tif]

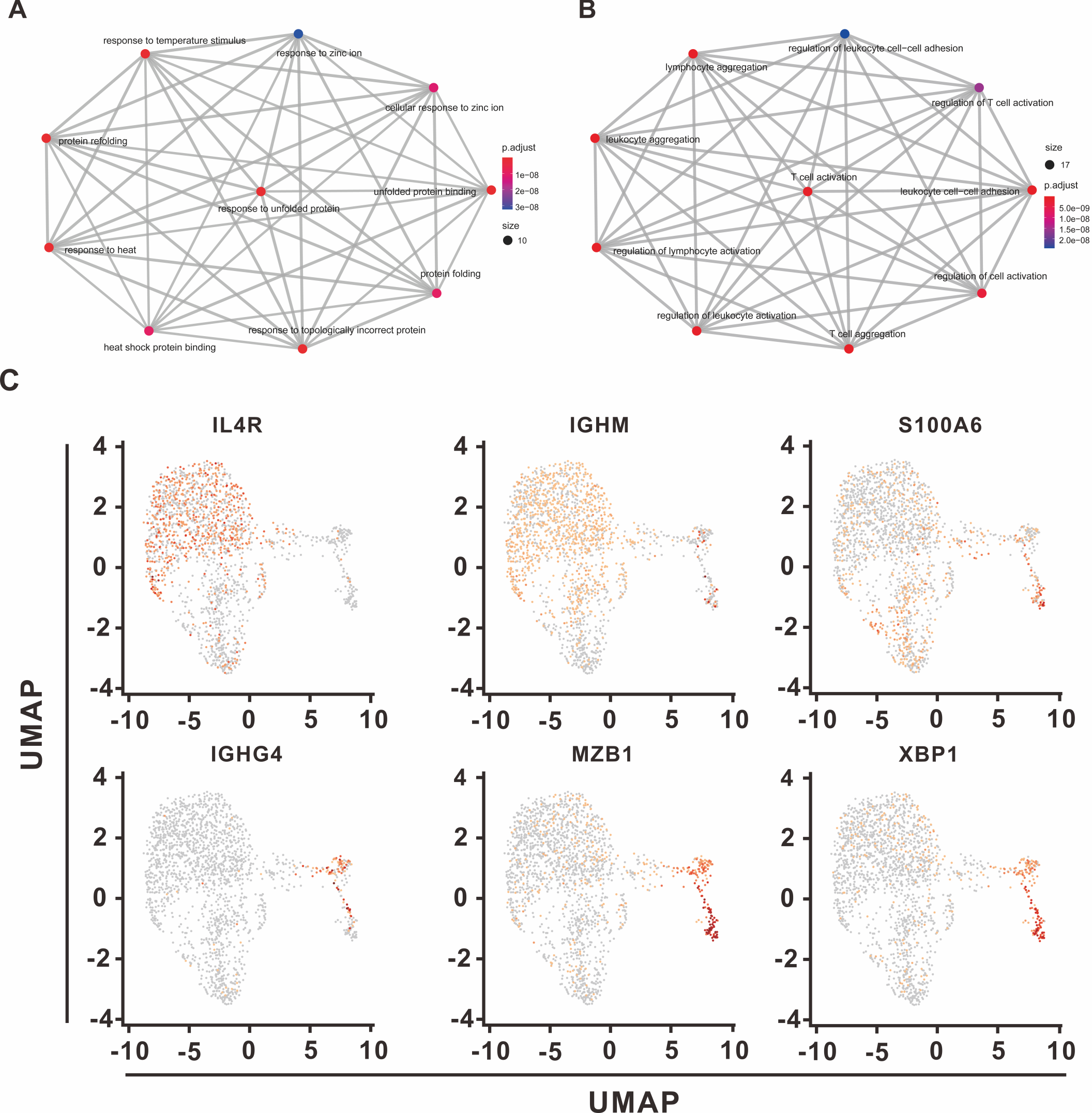

Supplement: Supplementary file 2 — Additional file 2. Fig. S2 (A) The GO enrichment analysis of CD8 + Teff subclusters C1-CD8 shows enriched pathways. (B) The GO enrichment analysis of CD8 + Teff subcluster C4-CD8 shows enriched pathways. (C) UMAP plot of the B cell subclusters annotated by some marker genes [file 12967_2022_3424_MOESM2_ESM.tif]
